# Tumor‐Tissue Boundaries as Instructive Interfaces in Breast Cancer Cell Invasion

**DOI:** 10.1002/advs.202509249

**Published:** 2025-09-11

**Authors:** Cornelia Clemens, Thomas Zerjatke, Andrine Frank, Hannah Trampert, Nataliia Kotsiuba, Ingmar Glauche, Tilo Pompe

**Affiliations:** ^1^ Institute of Biochemistry Leipzig University 04103 Leipzig Germany; ^2^ Institute for Medical Informatics and Biometry TU Dresden 01307 Dresden Germany

**Keywords:** breast cancer cells, cell migration, extracellular matrix interfaces, instructive phenotype switching

## Abstract

The ability of metastatic cancer cells to invade distant tissues requires them to cross a variety of tissue boundaries, each posing distinct structural and biochemical challenges. In particular, the boundary between dense, extracellular matrix (ECM)‐rich tumor tissue and surrounding stromal tissue is associated with phenotypic changes in MDA‐MB‐231 breast cancer cells following transmigration, including increased invasiveness and aggressiveness. It remains unclear whether this transition arises from selective, permissive filtering of pre‐existing subpopulations, such as cancer stem cells, or an instructive response of the entire cell population. Here, by combining single‐cell migration analysis, heterogeneity analysis of cell proliferation, and computational modeling, it is demonstrated that tumor‐tissue boundaries act as instructive interfaces. Using an established 3D fibrillar collagen I matrix model of interfaces, it is shown that all cells can transmigrate the interface with no evidence of selective filtering of subpopulations. Proliferation heterogeneity remains unchanged between transmigrated and non‐transmigrated cells, further supporting an instructive mechanism. Simulations confirm that the interface instructively modulates cell behavior. These results indicate that tissue boundaries can reprogram cancer cell phenotypes, representing a potentially targetable mechanism in metastatic progression.

## Graphical abstract

Tumor‐tissue boundaries reprogram metastatic breast cancer cells, enhancing invasiveness through an instructive, not permissive, mechanism. Using a 3D collagen I‐based tumor‐tissue interface model, single‐cell analysis, and simulations, this study shows that all cells can transmigrate without selective filtering, highlighting a targetable process in metastatic progression.

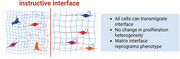

## 1 Introduction

Cancer is a multifaceted disease characterized by genetic and phenotypic diversity. Breast cancer, like many other malignant diseases, develops through a complex interplay of genetic mutations and environmental influences, which together determine the progression and the metastatic spreading of the cancer. Metastasis refers to the multi‐step process by which cancer cells detach from the primary tumor, invade surrounding tissues, enter the bloodstream or lymphatic system, and colonize distant organs, leading to the formation of secondary tumors.^[^ ^1^, ^2^ ^]^ Central to this process is the accumulation of somatic aberrations in the tumor cells.^[^ ^3^, ^4^ ^]^ These genetic alterations promote uncontrolled proliferation and can enhance the metastatic potential of tumor cells.^[^ ^5^ ^]^ One prominent example is mutations in the *TP53* gene, which frequently result in gain‐of‐function, promoting uncontrolled cell proliferation and enhancing tumor aggressiveness across multiple cancer types.^[^ ^6^ ^]^ The stochastic accumulation of mutations, driven by genomic instability, generates genetic diversity within the tumor, leading to the emergence of genetically and phenotypically distinct subclones.^[^ ^7^, ^8^ ^]^ This genetic and phenotypic heterogeneity emphasizes the adaptability and progression of the tumor, as well as posing a major challenge to effective treatment. Subclonal diversity can drive resistance to therapies, either through the expansion of preexisting resistant subclones or the emergence of new subclones driven by selective pressures imposed by treatment.^[^ ^9^ ^]^ In this context, cancer stem cells have been discussed in the literature as a highly aggressive subpopulation and a major source of recurrence of tumors after initial therapy.^[^ ^10^ ^]^ Understanding how tumor cell heterogeneity evolves during tumor progression and the metastatic cascade is essential, as it represents a hallmark of cancer that complicates both diagnosis and treatment.^[^ ^5^ ^]^ Moreover, increased tumor heterogeneity has been associated with worse clinical outcomes for patients.^[^ ^11^, ^12^ ^]^

Notably, beyond the genetic and phenotypic diversity within the tumor cells themselves, the tumor microenvironment (TME) undergoes extensive molecular and structural evolution during cancer progression. The TME consists of extracellular matrix (ECM) macromolecules that provide structural support, along with cellular components such as fibroblasts and immune cells, which contribute to the overall function of the TME.^[^ ^13^ ^]^ This evolving microenvironment not only drives further heterogeneity and promotes carcinogenesis but also plays a crucial role in tumor cell invasion and metastasis.^[^ ^14^ ^]^ One key aspect of this evolution is the increasing mechanical and architectural tissue heterogeneity. Many native tissues, including dermis, cartilage and wounded or regenerating tissues, exhibit spatially distinct zones that differ in ECM organization, density, and stiffness.^[^ ^15^ ^]^ In the context of cancer, zonal differences arise between tumor and surrounding tissues, forming discrete interfaces with sharp contrasts in ECM density.^[^ ^16^ ^]^ These structurally heterogeneous interfaces are increasingly recognized as regulators of invasive cell behavior and may significantly influence metastatic dissemination.^[^ ^15^, ^17^, ^18^, ^19^ ^]^ Progressive stiffening and densification of the tumor tissue are caused by increased deposition of components of the ECM, cross‐linking and realignment of matrix fibers.^[^ ^20^ ^]^ Among ECM components, collagen type I plays a central role in tumor stiffening, with its quantity, alignment, and cross‐linking profoundly influencing the biomechanical properties of the TME.^[^ ^13^ ^]^ These mechanical and structural changes within the TME shape tumor cell behavior through bidirectional communication between the cancer cells and the ECM.^[^ ^21^, ^22^ ^]^ Cells sense and respond to the altered physical properties of their microenvironment by modulating gene expression and remodeling their surroundings, creating a feedback loop that drives malignancy and promotes tumor aggressiveness. For instance, studies have demonstrated that collagen matrix properties, such as fibril diameter and cross‐linking, directly influence the migration and clustering behavior of breast cancer cells.^[^ ^23^, ^24^, ^25^, ^26^ ^]^ Furthermore, increased collagen density has been associated with increased invasive properties of tumor cells originating from solid tumors.^[^ ^27^ ^]^ Such interactions not only promote invasive phenotypes but also contribute to ECM remodeling events.^[^ ^28^ ^]^ As these processes continue to reshape the TME, they lead to the formation of well‐defined ECM interfaces at tumor boundaries, where contrasting biomechanical and compositional properties between tumor and healthy tissue become apparent.^[^ ^16^, ^29^ ^]^ These interfaces function as a first topological and mechanical discontinuity along the metastatic cascade, which invasive tumor cells have to cross to successfully invade the surrounding tissue.

Previous studies have established a biomimetic ECM model of the boundary between tumor and the healthy surrounding tissue to investigate breast cancer cell behavior during the early stages of metastasis as they transmigrate across tumor‐tissue interfaces.^[^ ^15^, ^17^ ^]^ The model used a design with two compartments, where two different 3D fibrillar collagen I matrices with distinct fibril densities were reconstituted through a sequential fibrillation process, creating a sharp interface of two different matrices. It was shown in several studies that MDA‐MB‐231 breast cancer cells embedded in a denser matrix compartment transmigrate across the interface into an open matrix, resulting in a distinct and significant phenotype switch toward a more invasive and aggressive behavior. Upon interface transmigration, these cells exhibited a transition from random to more directed migration, an increased proliferation and an enhanced chemoresistance toward doxorubicin.^[^ ^15^, ^17^, ^18^ ^]^ Importantly, this switch in phenotype was demonstrated to be not a consequence of invasion into a more open porous matrix or seeding cells therein, but was specifically triggered by the act of crossing the sharp structural discontinuity of the matrix interface itself. A dependence on altered matrix stiffness (or so‐called ‘durotaxis’) or chemotaxis within oxygen gradients was excluded as a trigger, too.^[^ ^15^ ^]^ Furthermore, transcriptomic analysis revealed a specific upregulation of gene clusters associated with metastasis in cells with changed phenotype.^[^ ^18^ ^]^ Increased DNA damage and mechanotransductional misregulation, driven by strong localized contractile forces during transmigration, were recently identified as potential mechanisms underlying these phenotypical changes.^[^ ^18^, ^19^ ^]^ Notably, while MDA‐MB‐231 cells typically exhibit higher invasive behavior in denser collagen I matrices,^[^ ^25^ ^]^ the reported phenotypical changes were absent in cells transmigrating across defined interfaces from open into dense matrices. Recent studies showed that open‐to‐dense transmigration does not induce changes in migratory behavior, morphology, proliferation, chemoresistance or gene expression signature, nor does it trigger mechanical stress responses or mechanotransductive signaling.^[^ ^18^, ^19^ ^]^

While these findings highlighted the profound impact of the structural properties of matrix interfaces and mechanical and biological stress on tumor cells during transmigration of tumor‐tissue boundaries, they also raised questions about whether the interface instructs phenotypic changes on the full cell population of breast cancer cells in the initial compartment or permissively filters a pre‐existing subpopulation with more invasive, aggressive or genetically instable properties. For instance, such pre‐existing and more aggressive subpopulations are discussed as cancer stem cells.^[^ ^30^ ^]^ Hence, this study builds on the preceding observations and asks whether the phenotypical change of breast cancer cells during transmigration of dense‐to‐open matrix interfaces arises from a selective permissive filtering of specific pre‐existing subpopulations or an instructive change of all cells of the initial population. We used the established MDA‐MB‐231 breast cancer cell line and the biomimetic tumor‐tissue interface model based on 3D fibrillar collagen I compartments for single‐cell migration analysis and cell counting in both compartments, as well as heterogeneity analysis of cell proliferation prior to and after transmigration. Results of permissive and instructive scenarios in corresponding simulations of cell behavior were compared to the cell culture experiments. Our results strongly support an instructive function of matrix interfaces as regulators of breast cancer cell behavior, providing valuable insights into the mechanisms by which ECM topology modulates tumor invasiveness and metastasis.

## 2 Results

### 2.1 Topological Features of Engineered Collagen I Matrix Interfaces and Their Functional Impact on Transmigrating Breast Cancer Cells

Alterations in the ECM, including changes in its structure, composition, and stiffness, have been linked to the progression and spread of cancer, directly influencing the metastatic cascade.^[^ ^31^ ^]^ During tumor progression, extensive matrix remodeling results in the formation of distinct interfaces between dense, stiff tumor tissue and the softer, more porous surrounding healthy tissue, posing a challenge for invasive breast cancer cells. Prior research has shown that as MDA‐MB‐231 breast cancer cells transmigrate across well‐defined collagen I‐based matrix interfaces. Specifically, when transmigrating from a dense to a more porous matrix, they undergo significant phenotypic changes, acquiring a more aggressive phenotype, including changes in gene expression, invasiveness, proliferation and chemosensitivity. In particular, it was found that the sharp increase in porosity, independent of matrix stiffness, induces this phenotype switch, emphasizing the importance of the structural discontinuity at the interface. Importantly, this effect is not a result of simple MDA‐MB‐231 breast cancer cell invasion in a more porous collagen I matrix, but a result of transmigrating a sharp topologically distinct matrix interface, as discussed in the introduction. Additionally, matrix interfaces with reversed configuration, where cells migrate from a porous into a denser matrix, did not result in any changes in cellular phenotype or gene expression.^[^ ^15^, ^17^, ^18^, ^19^ ^]^ This highlights the unique and significant role of tumor‐tissue interfaces in the early stages of breast cancer cell metastasis, where the cells exhibit phenotype switching in response to the matrix interface's topological properties. However, it raised the question of an instructive influence of the matrix interfaces on the full cell population or a permissive filtering of pre‐existing, more aggressive subpopulations.

**Figure 1:**
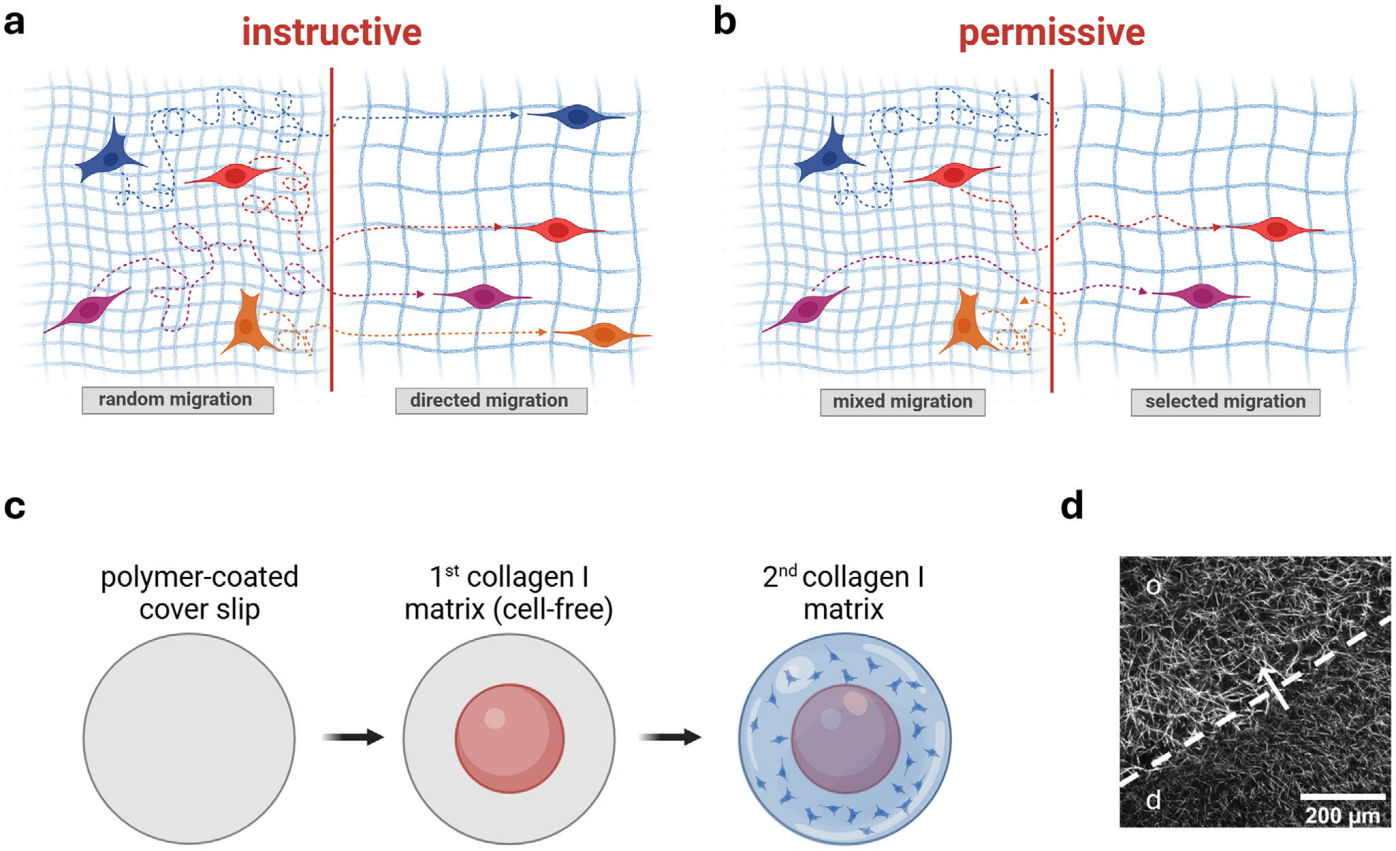
In vitro model recapitulating the sharp increase in matrix porosity at tumor‐tissue interfaces. a,b) Schematic representation of an (a) instructive phenotypical change induced by the matrix interface in contrast to a (b) permissive matrix interface. Different colors indicate different subpopulations within the full cell population. The dashed lines depict the proposed migration trajectories before and after transmigration across the interface. Created in BioRender. Clemens, C. (2025) https://BioRender.com/jlpcfu0 c) Experimental setup for generating collagen I matrix interfaces via sequential fibrillation, with MDA‐MB‐231 breast cancer cells embedded in the 2^nd^ and dense compartment. Created in BioRender. Clemens, C. (2025) https://BioRender.com/4rqw7hf d) Confocal laser scanning microscopy image of an in vitro matrix interface stained with TAMRA‐SE.

We now hypothesize that the distinct matrix interfaces between a tumor and a healthy tissue *instruct* cells to change their phenotype during transmigration (**Figure** **1**a). In this process, each cell that reaches the interface can transmigrate the interface and thereby transition to a state of elevated metastatic potential, for example, showing highly directed migration and proliferation (see also animation in Video S1, Supporting Information). This hypothesis is supported by earlier findings regarding the molecular mechanisms driving the phenotypical changes. Asymmetric forces that act on transmigrating cells during interface crossing, due to the different porosities of the adjacent matrix compartments, disturb mechanotransduction signaling, including nuclear integrity, further influencing the cellular phenotype through alterations in gene expression.^[^ ^18^, ^19^ ^]^ An alternative hypothesis suggests a permissive interface, where the tumor‐tissue boundary does *permissively* select cells, allowing only subpopulations with pre‐existing metastatic potential, like cancer stem cells, to pass through the matrix interface (Figure 1b; and Video S1, Supporting Information). Specifically, the highly invasive breast cancer cell line MDA‐MB‐231 has been shown to exhibit significant genetic and phenotypic heterogeneity, with some subpopulations expressing cancer stem cell markers in varying proportions.^[^ ^32^, ^33^ ^]^ In addition to their high self‐renewal capacity, cancer stem cells exhibit increased proliferation, chemoresistance, and motility.^[^ ^30^ ^]^ Thus, cancer stem cells may represent a distinct subpopulation of an initially larger cell population capable of crossing matrix interfaces and showing an overall more invasive and aggressive phenotype upon interface crossing that aligns with the observed results in cell experiments.

In this study, our aim was to prove (or disprove) the above hypothesis, that tumor‐tissue boundaries act as instructive interfaces on transmigrating breast cancer cells. To achieve this, we used the previously established 3D collagen I matrix interface model.^[^ ^15^, ^18^, ^19^ ^]^ This model consists of two compartments of fibrillar collagen I matrices with differing porosities reconstituted using a sequential fibrillation strategy (Figure 1c). As a result, well‐defined matrix interfaces are formed, separating a dense from a porous collagen I matrix, thereby mimicking an interface between a tumor and a healthy tissue (Figure 1d). Topological analyses, as described by Franke et al.,^[^ ^34^ ^]^ revealed a mean porosity of ≈4 µm for the dense matrix (‘d’), while the more open porous matrix (‘o’) had a porosity of ≈5 µm. The mean fibril diameter remained constant for all matrices at ≈0.95 µm (Figure S1, Supporting Information). The initial cell population was seeded in the dense compartment, formed in the 2^nd^ fibrillation step. In the following text, transmigration of cells from a dense into a porous matrix across a defined interface is abbreviated as ‘d→o’. Cells that did not cross the interface and stayed in the dense compartment are called ‘non‐transmigrated’ (nt), and the cells that crossed the interface are termed ‘transmigrated’ (t).

### 2.2 Characterization of Breast Cancer Cell Migration at Matrix Interfaces

**Figure 2:**
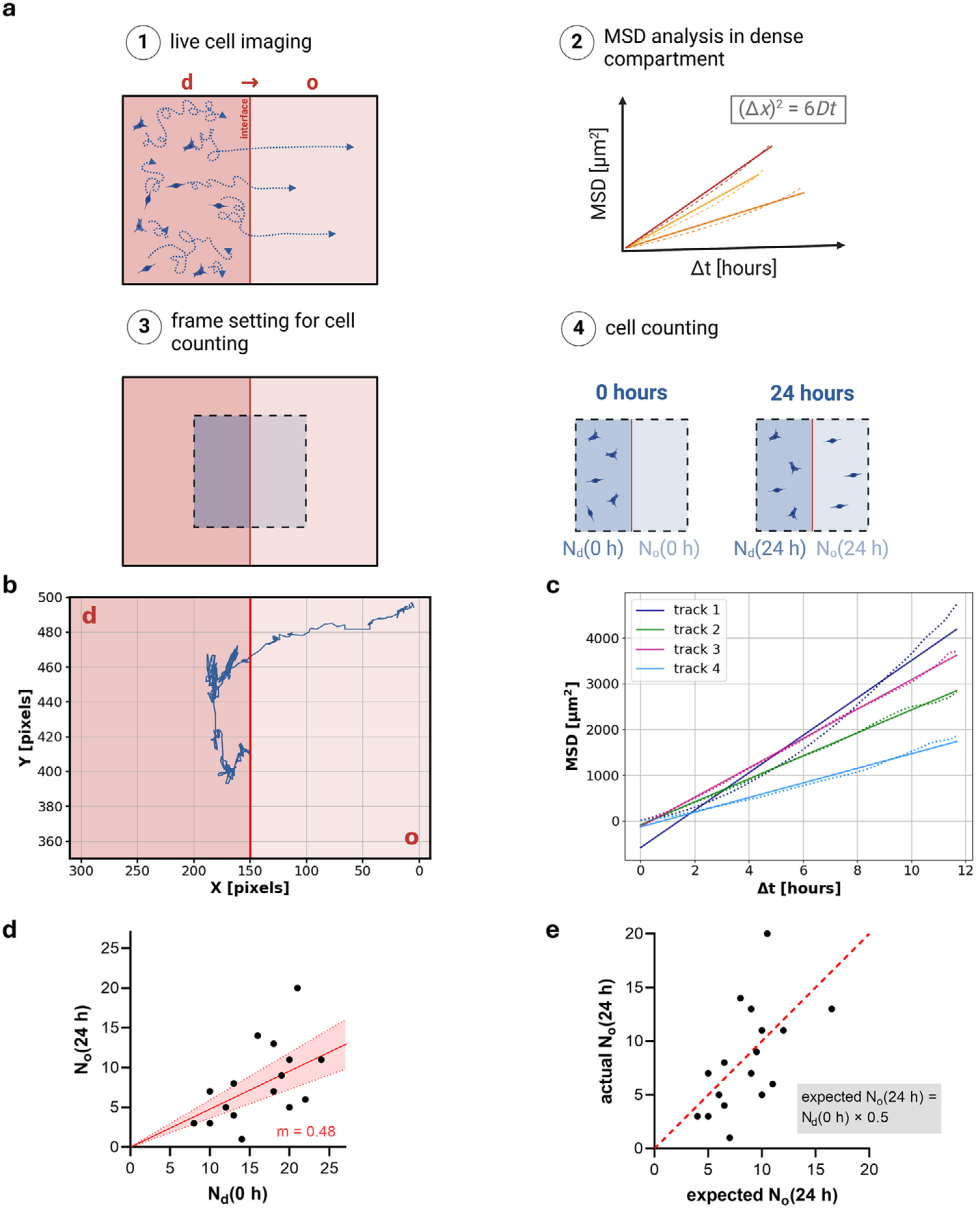
Single‐cell migration analysis using mean squared displacement (MSD) analysis and statistical assessment of cell numbers in proximity to the matrix interface. a) Schematic illustration of the analysis of live cell tracking data: 1) Imaging of MDA‐MB‐231 cells at the d→o matrix interface for 7 days; 2) MSD analysis in 3D to extract the diffusion coefficient D; 3) Setting a volume for cell counting in live cell imaging z‐stacks in both compartments next to the interface; 4) Cell counting within the defined frame at time points 0 and 24 h in both the dense (N_d_) and open (N_o_) compartments (N_d_(0h), N_o_(0h), and N_d_(24h), N_o_(24h)). Created in BioRender. Clemens, C. (2025) https://BioRender.com/ii3j194 b) Representative coordinates of an individual cell's track before and after transmigration, projected in x‐y plane. The interface is indicated by a red line. 1 pixel = 1.253 µm. c) Representative MSD results for 4 individual cells, plotted as a function of time interval ∆t. The maximum ∆t was 12 h. The dashed line represents the MSD, while the solid line shows the linear regression. d) Comparison of the cell numbers in the dense (N_d_(0h)) and open (N_o_(24h)) compartments at 0 and 24 h, with a linear regression (including a confidence interval of 95%) indicating the slope of m=0.48 (*n* = 17).

Understanding how breast cancer cells migrate at tumor‐tissue interfaces is critical to determine whether these interfaces instruct phenotypical changes or merely act as permissive barriers. To investigate this, single‐cell migration of MDA‐MB‐231 cells at d→o interfaces was characterized. By time‐lapse imaging of living MDA‐MB‐231 cells within the 3D matrix interface model for 7 days, their migration in proximity to the interface was tracked and analyzed on a single‐cell level (**Figure** **2**a,b; Figure S2a, Supporting Information). Quantification of migration metrics showed a significant increase in migration directionality after transmigration, while the migration speed remained constant at ≈0.3 µm min^−1^ (Figure S2a,c, Supporting Information), in agreement with previous results.^[^ ^15^, ^35^ ^]^ These observations indicate that the matrix interface, as a topological discontinuity, influences cell motility patterns from a random migration phenotype to a more directed cell migration without altering migration speed.

To determine whether all cells that reach the matrix interface are capable of crossing it or whether the interface selectively hinders certain cells lacking specific phenotypic properties, we estimated the number of cells able to reach the matrix interface within a defined time interval of 24 h. To establish this estimate, we developed a quantitative workflow based on the single‐cell tracking results (Figure 2a). Therefore, we used two assumptions, based on our experimental observations as reported above: 1) Cells exhibit in the first (dense) compartment a random migration characteristic. This allows to apply mean squared displacement (MSD) analysis of single‐cell tracks to assess average movement of migrating cells similar to a diffusion front.^[^ ^36^ ^]^ 2) Cells that successfully transmigrated into the open compartment adopt a directed migration pattern, minimizing their chance of crossing the matrix interface back into the dense compartment.

Based on these assumptions, we first analyzed the MSD of cells in the dense compartment to quantify their random migration behavior. To avoid bias from limited tracking durations or excessive noise in long‐term measurements, an appropriate maximal Δ*t* was identified using a snipping analysis. Therefore, the linear regression quality of MSD of individual cell tracks with varying maximum Δ*t* was assessed. The optimal fit (maximal *R* ^2^) as well as a plateau of the slope value within individual cell tracks were achieved for a maximum Δ*t* of 12 h (Figure S3, Supporting Information). With this optimized Δ*t*, final MSD analysis of single‐cell tracks (over a total imaging time of 72 h) was performed, revealing a mean diffusion coefficient of 0.75 ± 0.38 µm^2^ min^−1^ for MDA‐MB‐231 cells migrating in dense collagen matrices (Figure 2c; Figure S4, Supporting Information). This value aligns with previously reported diffusion coefficients for MDA‐MB‐231 cells in comparable 3D environments, reinforcing the reliability of our approach.^[^ ^35^ ^]^

To compare how many cells can reach the matrix interface by random migration within 24 h and can be actually found in the open compartment, we calculated the number of cells randomly migrating out of the dense compartment into the open compartment within 24 h *N*_o_(24h). Using an approach of cell diffusion out of a homogeneously populated half‐space with an absorbing boundary, we revealed the survival function *S*(*x*,*t*) and integrated (1 ‐ *S*(*x*,*t*)) over the half‐space (Section S1, Supporting Information). The absorbing boundary condition is motivated by our observation of the changed migratory phenotype after transmigration with a directed migration away from the interface. Using this approach, the determined diffusion coefficient of the MSD analysis, and the counted cell number within a box size of 156 µm perpendicular to the interface (x‐direction), we reveal:

(1) $$N_{o}\left(24h\right)=\frac{37\;\mu m}{156\;\mu m}N_{d}\left(0h\right)=0.23\cdot N_{d}\left(0h\right)$$

for the number of cells that could have left the dense compartment and will be found within the open compartment after 24 h.

Cells in similar volumes of both compartments (xyz: 156 µm × 156 µm × 250 µm) next to the interface were counted at 0 h and 24 h. Pre‐localized cells in the open compartment at time point 0 h can be assumed to leave the compartment within 24 h, because of the directed cell migration character and the measured migration speed (see above), hence, do not influence the cell counting results.

As the MDA‐MB‐231 cells exhibit a proliferation constant of 2 within 24 h, see also next section, a cell doubling has to be roughly assumed for cells leaving the dense compartment or being within the open compartment after transmigration. With this fact, we result at the final expectation that 46% of cells in the dense compartment at 0 h (*N*_d_(0h)) might be found after 24 h in the open compartment (*N*_o_(24h)), within our primary hypothesis that no permissive selection occurs at the interface. Since the migration experiments were conducted over a short duration of 24 h, small differences in cell proliferation due to changed phenotype (see also next section) do not strongly bias the cell counting.

The results show that the cell number in the open compartment at time point 24 h linearly scales by a factor 0.48 to the cell number in the dense compartment at 0 h (Figure 2d). This finding impressively agrees with the hypothesis of an instructive interface, where all cells of the population can transmigrate the matrix interface, as one would expect 46% of the initial cell number to be counted in the open compartment after 24 h, and we find 48% (Figure S5, Supporting Information).

In sum, the single‐cell migration analysis at matrix interfaces shows that the matrix interface does not act as a physical barrier to cell migration, neither restricting overall cell movement nor selectively permitting only cells with a possibly inherent higher migratory directionality to transmigrate. Instead, the findings support the hypothesis that the matrix interface induces intracellular signaling, altering the migratory behavior, leading to phenotypical changes upon transmigration in an instructive rather than permissive manner.

### 2.3 Heterogeneity in Proliferation of MDA‐MB‐231 Cells before and after Transmigration Across Biomimetic Tissue Interfaces

The triple‐negative cell line MDA‐MB‐231 is known for its inherent genetic and phenotypic heterogeneity.^[^ ^37^, ^38^ ^]^ This variability includes differences in migratory and proliferation behavior, which have been linked to distinct subpopulations within the cell line. Previous studies have demonstrated that these subpopulations can be sorted based on their migratory and invasive properties using collagen‐coated transwell assays or Matrigel‐coated membranes.^[^ ^33^, ^37^, ^39^ ^]^

Given the presence of these subpopulations, it is crucial to determine whether tumor‐tissue interfaces might act as permissive filters for distinct subpopulations with higher migratory and/or proliferative potential or act as instructive interfaces on the full population, as we hypothesize. To support our previous findings regarding unhindered cell migration across these interfaces, we investigated whether the transmigration of MDA‐MB‐231 breast cancer cells across matrix interfaces affects the heterogeneity of the population, specifically comparing cell proliferation before and after transmigration. We focused on heterogeneity in cell proliferation, as cell proliferation of MDA‐MB‐231 cells is known to be sensitively affected by subpopulation heterogeneity and is shown to be affected by transmigration of matrix interfaces.^[^ ^18^, ^33^ ^]^ A reduction in proliferation heterogeneity would point toward a permissive selection for certain subpopulations with specific traits. In contrast, unchanged heterogeneity would support the hypothesis of an instructive interface, influencing the entire population similarly.

**Figure 3:**
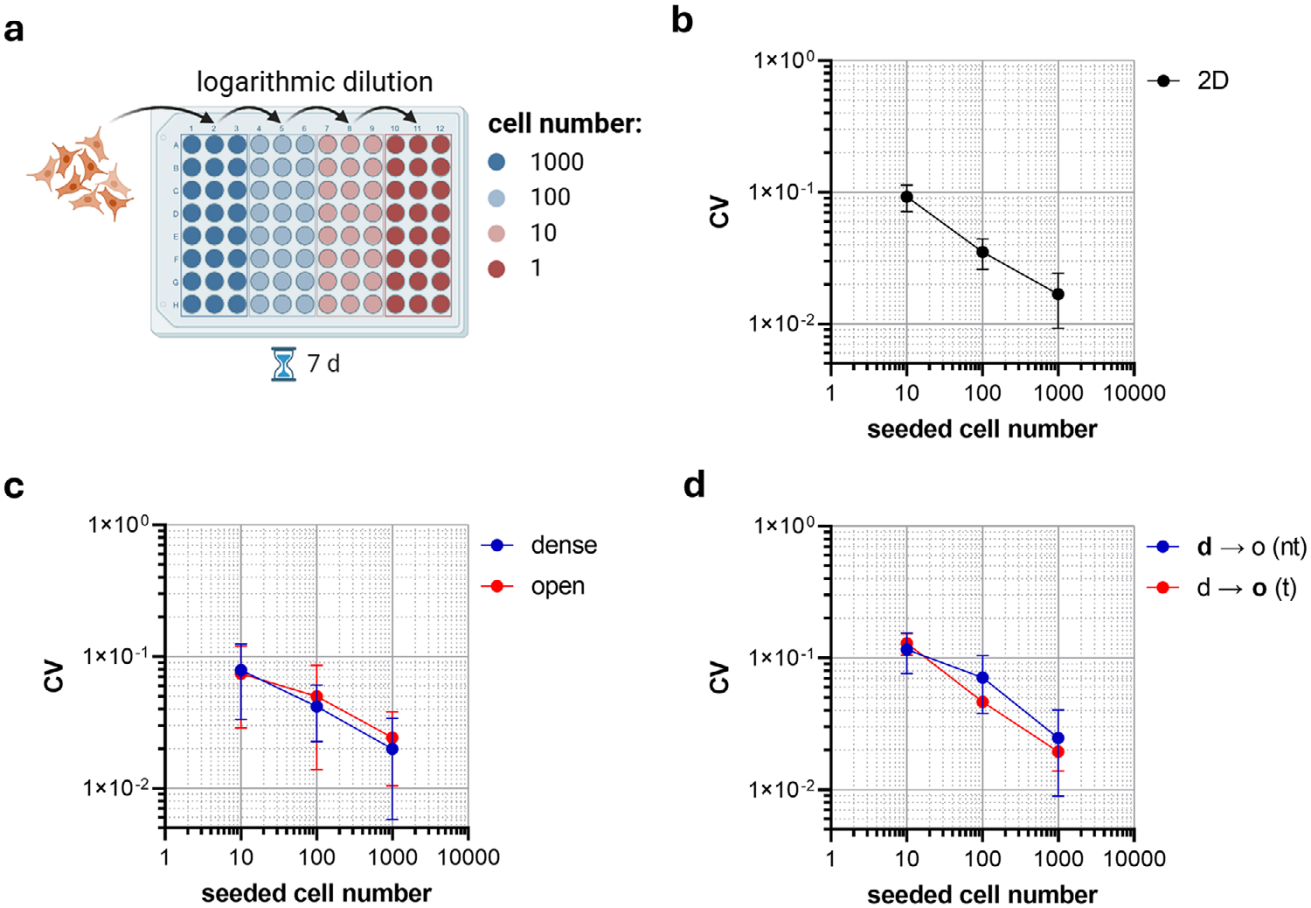
Quantification of cellular heterogeneity in proliferation using a 96‐well plate assay. a) Schematic representation of the logarithmic dilution series used to assess cellular heterogeneity in proliferation of MDA‐MB‐231 cells. Created in BioRender. Clemens, C. (2025) https://BioRender.com/qi6ryss b) Relationship between coefficient of variation (CV) and seeded cell number of MDA‐MB‐231 cells cultured before under standard 2D conditions in cell culture flasks. c) Comparison of CV vs. cell number of MDA‐MB‐231 cells cultured in homogeneous collagen I matrices with different porosities. d) Comparison of CV vs. cell number of MDA‐MB‐231 cells before and after transmigration across the d→o interface, assessing the impact of transmigration on population heterogeneity. Each experiment was performed in triplicates. Data are presented as mean ± SD.

To quantify cellular heterogeneity in proliferation, an assay was established in a 96‐well plate format (**Figure** **3**a; Figure S6a, Supporting Information). A logarithmic dilution series was used to create blocks of 24 wells, each containing a fraction of cells of the previous block down to the single‐cell level in the final block. After 7 days of incubation at cell culture conditions, proliferation was quantified using an ATP‐luminescence assay. Heterogeneity was evaluated by calculating the coefficient of variation (CV) of the results across the 24 wells for each seeded cell number. For the final block with on average one cell per well, data were not analyzed, as only a fraction of wells contained cells after the 7‐day incubation period.

The results of control experiments for cells cultivated under standard conditions in a cell culture flask showed that with increasing cell number, the CV significantly decreases (*p* = 0.004, N = 24), indicating less variability across wells with increasing cell number per well (Figure 3b; Figure S6b, Supporting Information). The decreasing CV for larger populations suggests that averaging effects reduce the influence of random fluctuations in subpopulation distribution. In contrast, at low cell numbers, stochastic effects play a more pronounced role. Specifically, in small populations, variations in the initial proportion of subpopulations are more likely due to random sampling, leading to differences in the ratios of cells with varying proliferation constants between wells. Over time, these differences can amplify, resulting in higher variability in proliferation and contributing to a higher CV. These results proved the feasibility of the assay.

Next, the heterogeneity of MDA‐MB‐231 cells cultivated in homogeneous dense and porous collagen I matrices was compared, also as a control. Previous studies have shown that matrix properties can directly influence cellular phenotype and increase cancer heterogeneity. For example, differences in matrix elastic modulus in the range of kPa have been linked to malignant transformation and epithelial‐to‐mesenchymal transition.^[^ ^40^, ^41^ ^]^ In our study, the collagen matrices are much softer, with elastic modulus ≈100 Pa.^[^ ^15^ ^]^ Despite this lower stiffness, it is still possible that differences in matrix topology or even subtle variations in elasticity might influence cellular behavior and, consequently, heterogeneity. When examining the cell populations in both matrix densities, a decrease in the CV similar to the 2D control was observed with increasing cell number (p(d) = 0.02, p(o) = 0.01, N(d, o) = 24), indicating reduced variability across the wells with increasing cell number (Figure 3c). Importantly, no significant differences in CV were detected between the two matrix densities (*p* > 0.99, N = 24), suggesting that the overall heterogeneity of the MDA‐MB‐231 cell line is not influenced by the matrix density and elasticity.

Finally, the heterogeneity of proliferation of MDA‐MB‐231 cells was compared before and after transmigration across the d→o matrix interface. Contrary to the alternative hypothesis that the matrix interface might act as a permissive barrier, filtering subpopulations with specific proliferation characteristics, no significant differences in CV were observed between the two populations (*p* > 0.99, N = 24) (Figure 3d). Furthermore, CV similarly dropped with increasing cell number as observed in the control experiments. This finding suggests that the transmigration process does not alter the overall heterogeneity of the cell population, meaning that cells crossing the matrix interface do so independently of their proliferation characteristics. This result again supports that tumor‐tissue boundaries are instructive interfaces rather than permissive, allowing all cells of the population to pass equally. The unchanged cellular heterogeneity contrasts with scenarios where microenvironmental constraints impose selective pressures on migrating cells, emphasizing that in this case, the matrix interface does not introduce additional heterogeneity or selection biases within the population.

### 2.4 Simulation of Influence of Subpopulation Number and Diversity on Cellular Heterogeneity in Proliferation

To verify the findings of the cellular heterogeneity assay, which showed no changes in cellular heterogeneity across different sets of cell culture conditions (including cell culture flasks, different 3D collagen matrices, and after matrix interface transmigration), we aimed on further investigating how different levels of heterogeneity and subpopulation numbers would manifest in our assay by using simulation experiments.

**Figure 4:**
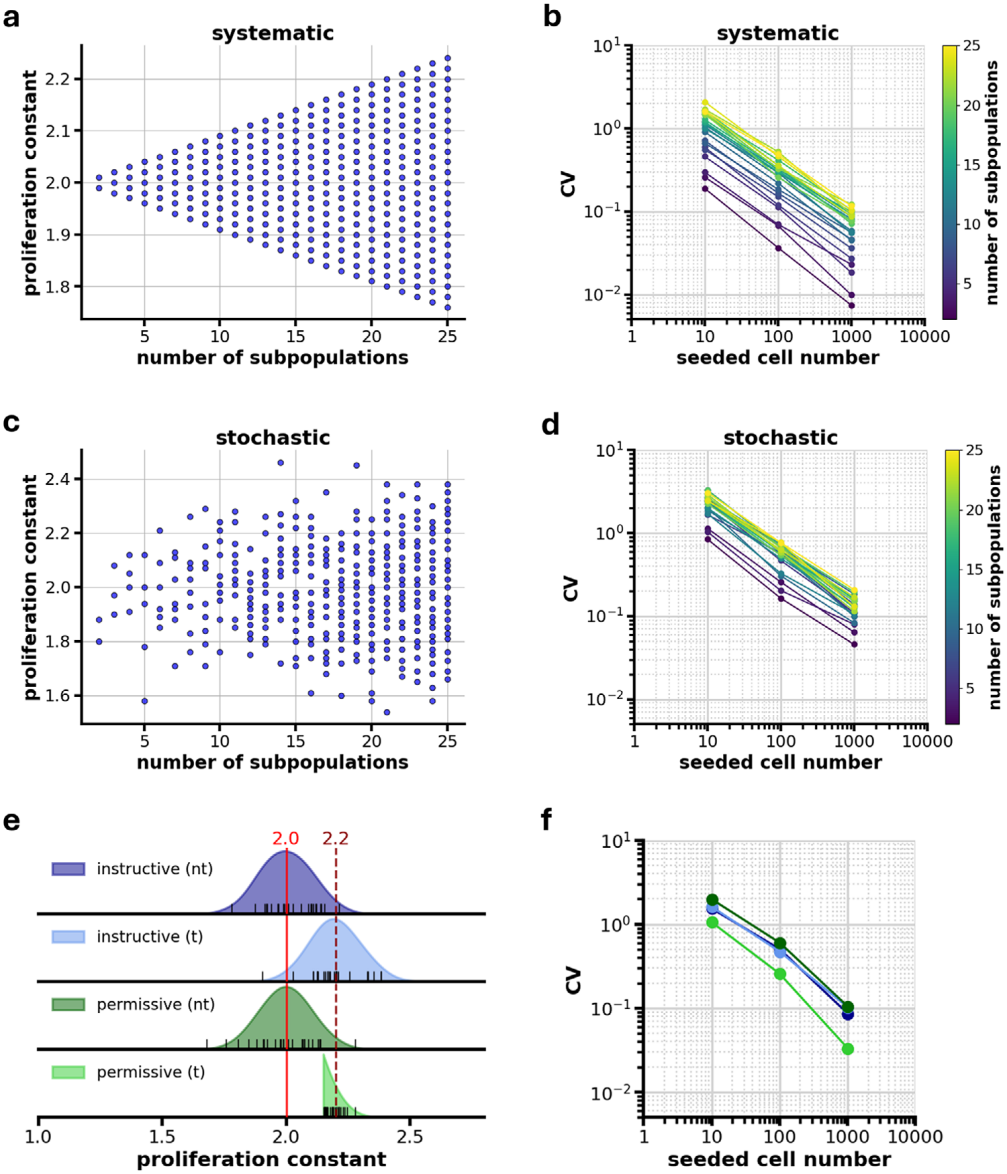
Simulation of the impact of subpopulation number and diversity on assessing cellular heterogeneity in proliferation. a) Proliferation constants in the 'systematic' model, showing how the range of proliferation constants broadens with increasing subpopulation number, with a fixed Δ of 0.05. b) Effect of subpopulation number on CV in dependence on cell number for the 'systematic' model. c) 'Stochastic' model of proliferation constants, where the proliferation constants are drawn from an underlying normal distribution by random sampling to reflect more biologically realistic variations. d) Effect of subpopulation number on CV in dependence on cell number for the 'stochastic' model. e) Proliferation constant distributions for cell populations at matrix interfaces before (nt – non‐transmigrated) and after (t – transmigrated) transmigration, for the instructive and permissive model. In the instructive model, a broad distribution (instructive (nt)) shifts from a mean proliferation constant of 2 to 2.2 (instructive (t)) upon transmigration. In the permissive model, the non‐transmigrated population (permissive (nt)) also exhibits a broad distribution centered ≈2. Here, transmigration acts as a selective process, filtering subpopulations with higher proliferation constants (R≥2.15), creating a transmigrated population enriched in cells with higher proliferation rates (permissive (t)). Black markers on the x‐axis represent 25 randomly sampled proliferation constants from these distributions. f) CV as a function of cell number for cell populations before and after transmigration, simulated using 25 subpopulations with the 25 randomly sampled proliferation constants from (e), showing possible effects of matrix interface transmigration on cellular heterogeneity. Data represent absolute values and are not averaged across multiple runs.

The simulations included various scenarios of cell population compositions, ranging from more homogeneous populations to populations with multiple distinct subclones, each exhibiting different proliferation characteristics. In a first set of simulations (*'systematic'*), (cancer) cell populations with an equal distribution of subpopulations in terms of cell number were assumed. Different numbers of subpopulations were tested ranging from *n *= 2 to 25. The proliferation constants *R*_n_ of these subpopulations were symmetrically distributed with a fixed Δ of 0.05 around a mean proliferation constant of 2, reflecting the reported doubling time of MDA‐MB‐231 cells within ≈24 h.^[^ ^42^, ^43^ ^]^ Consequently, an increase in the number of subpopulations resulted in a broader distribution of proliferation constants (**Figure** **4**a). Based on populations defined in such a manner, the logarithmic dilution series in the 96‐well plate assay was simulated by randomly sampling cells from the initial population and subsequently drawing from the corresponding wells in each dilution step. Assuming exponential growth, the cell numbers in each well were calculated after 7 days based on the assigned proliferation constants. This approach enabled the simulation of proliferation variability across different levels of cellular heterogeneity in the wells, offering insights into how subpopulation number influences assay results.

The simulation results confirmed the experimental data (Figure 3b,c), with CV significantly decreasing with increasing seeded cell numbers (*p* < 0.001, N = 24) (Figure 4b). Additionally, the simulation revealed that increasing the number of subpopulations led to an overall increase in CV across all seeded cell numbers (*p* < 0.001, N = 24). This result suggests that a higher heterogeneity within the population results in more pronounced variability in proliferation outcomes. In highly heterogeneous populations, differences in proliferation constants lead to higher well‐to‐well variability, even at higher cell numbers, which would be possible to observe in actual cell culture experiments.

To increase the biological relevance of the simulation, the approach to generate proliferation constants *R*_n_ was refined, by assuming a normal distribution centered around the mean proliferation constant (Figure 4c). In this modified model (*'stochastic'*), proliferation constants for each subpopulation *R*_n_ were randomly sampled from this distribution, better capturing natural variations in proliferation rates within heterogeneous cancer cell populations. This approach accounts for the fact that in real biological systems, proliferative differences among subpopulations are not necessarily evenly spaced but rather follow a more continuous distribution. Again, a decreasing CV with increasing cell number was found, similar to the systematic model of the simulation and the actual cell culture experiments. Although smaller in absolute numbers, the trend of increasing overall CV with a higher number of subpopulations was also found to be similar as in the systematic model (*p* < 0.001, N = 24) (Figure 4d). This behavior is expected as the variance of *R*_n_ in the stochastic model remained constant (determined by the normal distribution), in contrast to the systematic model, in which increasing the number of subpopulations led to a broader range of *R*_n_ values. This explains the slightly weaker dependence on the number of subpopulations in the stochastic model. Additionally, introducing an experimental error of 3% in the logarithmic dilution series to account for potential technical inaccuracies, for example pipetting errors, also led to a minor reduction in the effect size but did not alter the overall significance in respect to the dependence of CV on number of subpopulations (*p* < 0.001, N = 24) (Figure S6c, Supporting Information).

After verifying that the simulations show a similar dependence of CV on cell number as the experiment and can reveal a CV dependence on the number of subpopulations, we investigated how matrix interface transmigration influences cellular heterogeneity. We simulated two distinct scenarios that aimed to better reflect our experimental conditions and data, comparing the effects of an instructive versus a permissive interface. In both, the instructive and permissive models, the initial non‐transmigrated populations were assumed to have a broad, normal distribution of proliferation constants centered around a mean value of 2 (Figure 4e). This reflects a diverse population, with subpopulations exhibiting different proliferation behaviors. Proliferation constants for the non‐transmigrated and transmigrated MDA‐MB‐231 cell population were derived from previous studies, where a significant increase in proliferation after transmigration was reported (*R*(nt) = 2 and *R*(t) = 2.2).^[^ ^18^ ^]^ In the instructive model, all cells in the population are hypothesized to respond uniformly to the matrix interface upon transmigration, resulting in a coordinated increase in proliferation. It is assumed that this leads to a uniform shift of the proliferation constants of the entire population, with the distribution of proliferation constants shifting from a mean of 2.0 to 2.2. Thus, in this model, all cells undergo a similar phenotypic change in response to the interface, and the heterogeneity of the population remains constant. In the permissive model, only subpopulations with inherently higher proliferation constants (*R *≥ 2.15) are hypothesized to be selected upon transmigration by the interface and migrate across it. As a result, the transmigrated population represents a subset of the initial population, enriched for cells with higher proliferation constants. Consequently, the population is more homogeneous, consisting exclusively of cells with higher proliferation constants.

To model these scenarios, we randomly sampled 25 subpopulations from the initial distributions. These subpopulations were then used in the simulation of the heterogeneity assay (Figure 4f). The simulation results showed that in the instructive model, where all cells responded uniformly to the interface, the overall heterogeneity remained unchanged, resulting in no difference in CV between the non‐transmigrated and transmigrated populations. In contrast, the permissive model led to an overall reduction in CV after transmigration. This decrease in variability occurred because the matrix interface filtered for more rapidly proliferating subpopulations, creating a more homogeneous population.

This difference in CV versus cell number of the instructive and permissive model would be clearly visible in the actual cell experiments. As we do not find such a difference in the cell culture experiments (Figure 3b,c), the simulation results again support our hypothesis of tumor‐tissue boundaries to act as instructive interfaces.

Overall, the simulation experiments demonstrate that our assay on cellular heterogeneity in proliferation effectively captures variations in cellular heterogeneity, including number and spread of subpopulations. While experimental results revealed no significant differences in the trend of CV *vs*. cell number between the non‐transmigrated and transmigrated populations, the simulations showed that a higher number of subpopulations with varying proliferation constants leads to an increased CV. These findings align with the simulation's predictions, that in the case of an instructive interface with no changes in heterogeneity, no changes in CV are expected. Together, the results confirm that the transmigration across the matrix interface does not alter cellular heterogeneity and supports the hypothesis that the matrix interface acts in an instructive rather than permissive manner.

## 3 Discussion and Conclusion

Changes in ECM properties, such as increased collagen density and stiffness in the TME, play a crucial role in modulating tumor cell behavior, particularly at interfaces between tumor and healthy tissue, which serve as a critical site for dissemination of cells from the primary tumor. Previous studies using a biomimetic tumor‐tissue interface model based on fibrillar collagen I demonstrated that MDA‐MB‐231 breast cancer cells transmigrating across a sharp matrix interface, from a dense to a porous matrix, exhibited a more invasive and aggressive phenotype after transmigration.^[^ ^15^, ^18^ ^]^ This aggressive phenotype is marked by changes in migration patterns, such as enhanced directionality, as well as elevated proliferation. Notably, these studies have suggested that the d→o interface plays an important role in modulating tumor cell behavior, as differences in DNA damage and mechanotransduction signaling were observed in transmigrating cells directly at the matrix interface.^[^ ^18^, ^19^ ^]^ However, the precise mechanisms by which the matrix interface may either instruct phenotypic changes or permissively enrich pre‐existing subpopulations with increased invasive and therefore migratory and proliferative properties remained unclear. Importantly, such defined interfaces are not exclusive to tumors as they are a structural feature of many healthy tissues, including skin, cartilage, and regenerating wounds. Therefore, understanding how these topological transitions influence cell behavior may offer insight into a more general biological principle with implications beyond cancer.

To address this, we investigated two potential hypotheses, instructive and permissive matrix interfaces. The instructive hypothesis posits that the matrix interface alters cellular phenotype of all transmigrating cells by triggering mechanotransductional processes, leading to increased invasiveness and aggressiveness of all cells post transmigration. In contrast, the permissive hypothesis suggests that the interface merely acts as a selective filter, allowing only those cells with a certain pre‐existing characteristic, such as pre‐existing aggressiveness or potential to develop aggressiveness, to transmigrate across the matrix interface.

To distinguish between these mechanisms, we focused on investigating the impact of tumor‐tissue matrix interfaces on both migration and proliferation as markers for phenotype switching in the MDA‐MB‐231 breast cancer cell line upon interface transmigration. We first analyzed single‐cell migration characteristics at biomimetic tumor‐tissue interfaces and found no evidence of migrational hindrance or permissive filtering for MDA‐MB‐231 breast cancer cells. In the second set of experiments, we analyzed cellular heterogeneity with respect to proliferation, in combination with simulations of this assay. The simulation revealed that a permissive interface would result in a reduced heterogeneity by selectively enriching subpopulations with already higher proliferation and migration capacities. However, no significant differences in heterogeneity were observed between transmigrated and non‐transmigrated populations in our experiments. Hence, our findings on cell migration and proliferation heterogeneity suggest that the entire population undergoes a coordinated, instructed phenotypical change, rather than selective enrichment of aggressive subpopulations through the permissive function of the matrix interface. Therefore, our study proves the hypothesis of instructive properties of the biomimetic tumor‐tissue boundary.

Notably, the breast cancer cell line MDA‐MB‐231 is inherently characterized by a high heterogeneity.^[^ ^37^, ^38^ ^]^ Previous studies have highlighted the presence of distinct migratory subpopulations within MDA‐MB‐231 cells, where weakly migratory cells exhibited a higher metastatic potential, in part due to circulating tumor cell clustering and E‐cadherin expression.^[^ ^39^ ^]^ This fact also contradicts a hypothesis of a permissive filtering mechanism of a more aggressive subpopulation at d→o matrix interfaces, as we observed a highly migratory and invasive population after transmigration of the matrix interface. Furthermore, cancer stem cells are discussed to constitute a highly tumorigenic and therapy‐resistant subpopulation with increased metastatic potential. However, their overall abundance in breast cancer is relatively low,^[^ ^44^ ^]^ making it unlikely that cancer stem cells alone account for the observed phenotypic change during transmigration of the d→o matrix interface at purely permissive filtering constraints. Our simulations also indicate that a more pronounced heterogeneity would be required to account for the observed significantly increased proliferation when only a purely permissive selection is in place.

Altogether, our findings proof the hypothesis, that the d→o matrix interfaces modulate breast cancer cell behavior in an instructive manner and disprove a passive permissive filtering. This finding has important implications for understanding tumor progression and metastasis, as well as for developing therapeutic strategies that target ECM‐mediated cues. Since tissue interfaces are encountered repeatedly during metastasis and are also traversed by immune and regenerative cells in physiological contexts, our results suggest that interface topology may represent a broader regulatory mechanism in cell state transitions.

Future studies should aim to identify the molecular mechanisms underlying this instructive effect. Blocking studies targeting mechanotransduction pathways might assess the potential for therapeutic intervention. In addition to exploring this mechanism across different breast cancer cell lines, it will be important to investigate whether other cell types, such as immune cells, also exhibit interface‐driven phenotypic modulation. Such studies could reveal if matrix interfaces broadly contribute to guiding cell behavior in both pathological and physiological settings.

## 4 Experimental Section

### Reconstitution and Characterization of Collagen I Matrix Interfaces

3D collagen I matrices with defined interfaces separating compartments of contrasting porosities were prepared on glass coverslips (Ø 20 mm, VWR, Germany) following the previously published approach.^[^ ^15^, ^18^, ^19^ ^]^ After the surface modification of the glass coverslips with 3‐aminopropyltriethoxysilane (Roth, Germany), the coverslips were coated with 0.14% w/w poly(styrene‐*alt*‐maleic anhydride) (PSMA, MW 30,000 g mol^−1^, Sigma Aldrich, Germany). Subsequently, two collagen I solutions with different concentrations were prepared by mixing collagen I stock solution (3.9 mg mL^−1^, Advanced Biomatrix, US) with 0.02 N acetic acid (VWR) and 500 mm phosphate buffer (monosodium and disodium phosphate, pH 7.5, Sigma Aldrich) on ice. The final collagen I concentrations were set to 1.5 and 3.0 mg mL^−1^. To create a defined interface between two matrix compartments mimicking the tumor‐tissue boundary 50 µL of the first collagen solution was transferred on the coverslip and polymerized at 37 °C in a humidified chamber. After washing with phosphate buffer, 250 µL of the second collagen solution was layered on top of the first solution and polymerized accordingly.

Following the protocol by Franke et al. (2014)^[^ ^34^ ^]^ the topology of the collagen matrices was visualized using 50 µm TAMRA‐SE (VWR) staining and imaged via confocal laser scanning microscopy (LSM700) and a 40x immersion objective (both, Carl Zeiss Microscopy, Germany). Image stacks with a total size of 50 µm were recorded at 5 µm z‐intervals (resolution: 1024 x 1024 px, 0.16 µm px^−1^). Mean pore and fibril diameters were quantified using a home‐built MATLAB‐based image analysis tool.

### Cell Migration Experiments

The invasive breast cancer cell line MDA‐MB‐231 (DSMZ, Braunschweig; RRID: CVCL_0062) was cultured in DMEM (VWR) supplemented with 10% FCS (Merck, Germany) and 1% Zellshield (Minerva Biolabs, Germany) under standard conditions (37 °C, 5% CO_2_, 95% humidity). The cell line was confirmed to be free of contamination by the supplier. For the analysis of breast cancer cell migration across collagen I matrix interfaces, MDA‐MB‐231 cells were embedded in the dense compartment. To do so, cells were suspended in phosphate buffer and as described above mixed with collagen I solution and acetic acid to receive a final cell number of 10^5^ cells in the matrix compartment. After matrix reconstitution, the collagen interface matrices were placed in 12‐well plates, washed with warm 1x PBS and incubated with DMEM under standard conditions.

### Mean Squared Displacement‐Based Analysis of Single‐Cell Migration Prior Transmigration and Cell Counting for Transmigration Analysis within 24 h

For 3D migration studies, the interface matrices with embedded MDA‐MB‐231 cells were pre‐incubated for 24 h at 37 °C, 5% CO_2_, and 95% humidity and subsequently transferred to an inverted fluorescence microscope (Axio Observer.Z1, Carl Zeiss Microscopy) equipped with an incubation chamber. Over a 7‐day period, image stacks with a total size of 250 µm with a z‐interval of 5 µm were recorded every 10 min in transmission mode with a 10x dry objective at the matrix interfaces. Each image had a size of 692 x 520 px (resolution: 1.253 µm px^−1^).

Single‐cell tracking was performed manually using ImageJ's Macro MTrackJ.^[^ ^45^, ^46^ ^]^ The obtained xyz‐coordinates were used for mean squared displacement analysis (MSD) within the dense compartment (prior transmigration) using Python 3.13. To determine an appropriate maximal segment size Δ*t*, values ranging from 2 to 24 h were tested in a snipping analysis. For each track, MSD values were plotted against Δ*t*, followed by linear regression analysis. The slope of the regression line was used to determine D as a measure of diffusion coefficient, based on the relationship for 3D diffusion:

(2) $${\left(\Delta x\right)}^{2}=6D\cdot \Delta t$$

With this diffusion coefficient and an approach of cell diffusion out of a homogeneously populated half‐space with an absorbing boundary (Section S1, Supporting Information) the number of cells possibly transmigrating the matrix interface within 24 h was determined and compared it with the observed cell number. To do so, z‐stacks images next to the matrix interface were cropped for dense and open compartments separately using ImageJ^[^ ^45^ ^]^ and cells were counted in defined volumes of both compartments prior and after a 24 h interval of the 7‐day imaging period (see also Figure 2a). Specifically, the evaluated volume extended 312 µm along the x‐axis (with 156 µm perpendicular to the interface in each compartment), 312 µm in y‐direction parallel to the interface, and 250 µm in z‐direction (the full size of imaged z‐stacks).

Additionally, xyz‐coordinates were used to quantify migrational speed and directionality in each matrix compartment separately. Directionality was determined as the ratio of Euclidean distance to accumulated distance, resulting in a ratio between ‘1’ for highly directed migration and ‘0’ for a random migration mode.

### 96‐Well Plate Heterogeneity Assay

To analyze cellular heterogeneity, a 96‐well plate was loaded with 50 µL of 2.0 mg mL^−1^ collagen I per well and incubated at 37 °C in a humidified chamber for polymerization. Following polymerization, the matrices were washed twice with 1x PBS. The matrix interface compartments were then separated using tweezers and digested in a solution of 200 U mL^−1^ Collagenase IV (Life Technologies, US) in HBSS buffer with Ca^2+^ and Mg^2+^ (VWR) for 2 h at 37 °C after 7 days of cell culture. Following digestion, the resulting two cell suspensions with transmigrated (t) and non‐transmigrated (nt) cells were seeded into the prepared 96‐well plate.

Cells were seeded using a logarithmic dilution series. Initially, a cell suspension of 1.01 cells mL^−1^ was added to the first three columns of the well plate, resulting in 1,111 cells per well. A multichannel pipette was then used to perform serial dilutions across the plate, generating four blocks of 24 wells with decreasing cell counts of 1,000, 100, 10, and 1 cell per well. The assay cultures were then incubated under standard cell culture conditions for 7 days.

ATP concentration in the wells was analyzed using the *ATP Cell Viability Luciferase* assay (Sigma Aldrich) according to the supplier's instructions at the end of the 7‐day cell culture period. After addition of the assay's reaction mix, luminescence was measured in a microplate reader (Tecan Spark Multimode Microplate Reader, Switzerland) with a delay time of 5 min and a measurement duration of 1,000 ms. The coefficient of variation (CV) across 24 wells for each initial cell number was calculated to assess cellular heterogeneity.

### Computational Modelling of Cellular Heterogeneity

A computational simulation was developed to model the growth dynamics of heterogeneous cell subpopulations in the 96‐well plate format heterogeneity assay. The aim was to assess the impact of varying subpopulation numbers on overall heterogeneity and CV values.

In the first block of 24 wells, each well was seeded with 1,111 cells, randomly drawn from a pool, where cells were distributed equally among *n* subpopulations. Each subpopulation was assigned a specific proliferation constant (*R_n_*). A logarithmic dilution series was performed by randomly selecting cells from the corresponding well in the previous block, resulting in subsequent wells seeded with 1,000, 100, 10, or 1 cells. After a 7‐day incubation period, cell numbers were predicted using the formula:

(3) $$N\left(t\right)=\sum_{n}\left(N_{n,0}\cdot {R_{n}}^{t}\right)$$

*R_n_* represents the subpopulation‐specific proliferation constant, *N(t)* the total number of cells across all subpopulations after time *t* (in days), and *N_0_* the seeded/initial cell number. To quantify heterogeneity, the CV was calculated for wells seeded with the same initial cell number (**Table** **1**).

**Table 1:** Third‐Party Libraries Used under Python.

Library	Version	Source
Numpy	2.2.3	[47]
Matplotlib	3.10.0	[48]
Pandas	2.2.3	[49]
Statsmodels	0.14.4	[50]

### Statistical Analysis

Each experiment was carried out in triplicate, unless stated otherwise. Statistical significance was determined with a Kruskal‐Wallis or Mann‐Whitney test using GraphPad Prism 9 (GraphPad Software, USA) or Python 3.13 with the library statsmodels 0.14.4.^[^ ^50^ ^]^ The significance levels were set at *p* < 0.05 (*: *p* < 0.05; **: *p* < 0.01; ***: *p* < 0.001). Confidence intervals were set to 95% in respective regressions. Error bars represent standard deviation.

## Conflict of Interest

The authors declare no conflict of interest.

## Author Contributions

C.C. performed conceptualization, investigation, formal analysis, wrote the original draft, and edited the draft. T.Z. performed conceptualization and wrote, – reviewed, and edited the draft. A.F. performed investigation and formal analysis. H.T. performed investigation and formal analysis. N.K. performed investigation and formal analysis. I.G. performed conceptualization and wrote, reviewed, and edited the draft. T.P. performed Conceptualization, wrote the original draft, reviewed, and edited the draft.

## Supporting information



Supporting Information

Supplemental Video 1

## Data Availability

All data and home‐built MATLAB and Python scripts reported in this work are available from the corresponding author upon reasonable request. The MATLAB script for network topology analysis is freely available at https://git.sc.uni‐leipzig.de/pe695hoje/topology‐analysis.
