# Supplementary material for: Tumor‐Tissue Boundaries as Instructive Interfaces in Breast Cancer Cell Invasion
Source: Adv Sci (Weinh). 2025 Sep 11;12(44):e09249. doi: 10.1002/advs.202509249 (PMC12667463; doi:10.1002/advs.202509249)
Supplement: Supplementary file 1 — Supporting Information [file ADVS-12-e09249-s002.pdf]

Supporting Information

Tumor-Tissue Boundaries as Instructive Interfaces in Breast Cancer Cell Invasion

Cornelia Clemens, Thomas Zerjatke, Andrine Frank, Hannah Trampert, Nataliia Kotsiuba, Ingmar Glauche, Tilo Pompe*

Animation of Migration across 'Instructive' *vs*. 'Permissive' Interfaces

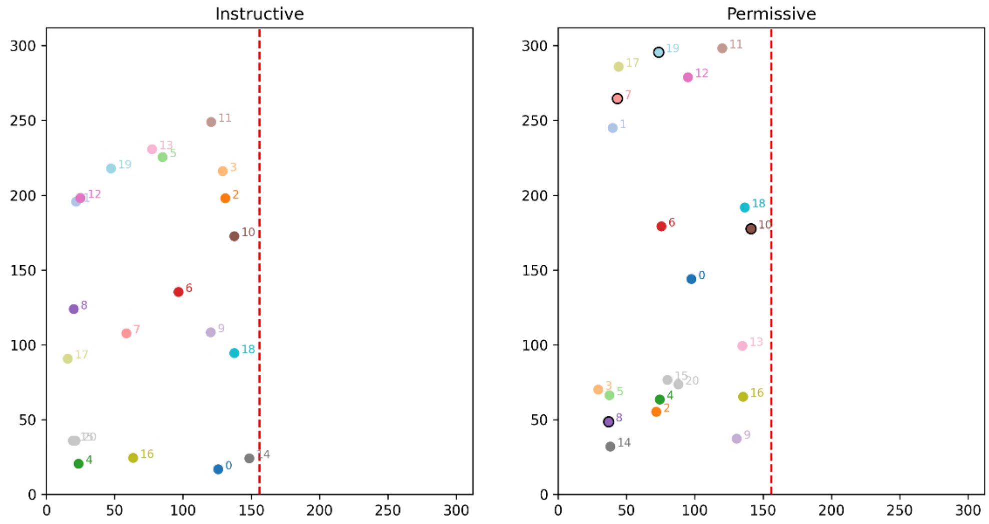

Video S1: *Animation of cell transmigration across biomimetic tumor-tissue interfaces under two distinct conditions:* 'instructive' *and* 'permissive'. *Each trajectory represents an individual cell, color-coded for distinction, over 144 time steps, with all cells starting in the left ('seeding') compartment.* Left*: In the 'instructive' condition, cells randomly migrating in the starting compartment cross the interface (red dashed line) and receive a directional bias toward the right as well as an increased proliferation after transmigration.* Right*: In the 'permissive' condition, cells already exhibit either random movement or to a small part a biased movement combined with increased proliferation (cells marked with a black outline) in the starting compartment, and randomly migrating cells are selectively restricted from transmigrating the interface.*

Animation of Cell Migration at Matrix Interfaces

To animate different cell migration behaviors, a computational model was implemented to demonstrate the movement of 20 individual cells on a 312 × 312 µm grid. The animation compared two distinct mechanisms: 'instructive' and 'permissive'. Cell movement was simulated in 10-minute intervals over 288 time steps, representing a 48-hour period, with cells starting in the left compartment, as the initial 'seeding' compartment. In the 'instructive' model, all cells initially exhibit a random migration. Additionally, cells that transmigrate the defined interface at x=150 µm receive a directional bias toward rightward movement. Furthermore, cells that had crossed the interface also adopted an elevated proliferation rate, implemented as a shortened division interval corresponding to a proliferation constant of 2.2, compared to a baseline of 2.0. This mimicked a context-specific increase in proliferation after interface transmigration. In contrast, the 'permissive' model employed a random migration mode, too, however, with an additional random directionality bias as well as an elevated proliferation rate applied to 20% of the cells. In this model, cells reaching the interface at x=150 µm maintained their initial migration characteristic, but, cells without the directionality bias were selectively

restricted from moving rightwards (transmigrating the interface). Mean speed (0.3 μm/min) was based on our measurements and other reports (Sapudom et al., Adv. Healthc. Mater. (2016)). The positions of all cells were tracked and visualized over time.

Topological Parameters of Collagen I Matrices and Matrix Interfaces

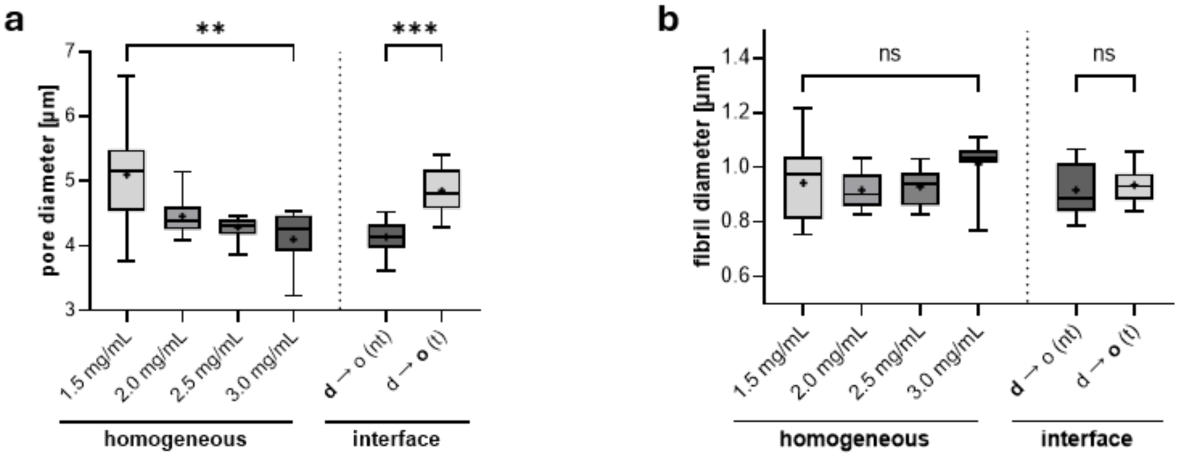

Figure S1*: Topological analysis of collagen I matrices and d→o matrix interfaces. Collagen I matrices were stained with TAMRA-SE and imaged via confocal laser scanning microscopy. An in-house MATLAB algorithm was employed to quantify key topological parameters: (a) mean pore diameter and (b) mean fibril diameter. Data are presented as box plots with a cross indicating the mean. Statistical significance was assessed using a Kruskal-Wallis test. Data were previously reported in Clemens et al., Biomater. Sci. 2025. The significance levels were set at $p < 0.05$ (*: $p < 0.05$; **: $p < 0.01$; ***: $p < 0.001$). n = 3.*

Quantitative Analysis of 3D Migration Trajectories of MDA-MB-231 Cells

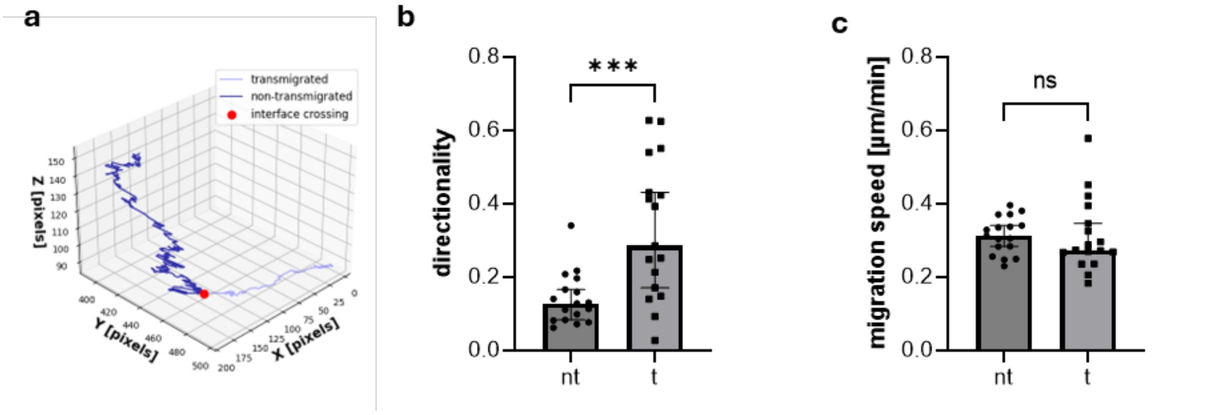

Figure S2: *Quantitative analysis of three-dimensional MDA-MB-231 cell migration trajectories. (a) Example of a three-dimensional cell track, with the red dot indicating the moment the cell crossed the interface into the open porous compartment. 1 px = 1.253 µm. (b) Directionality of cell migration of migration before and after transmigration across biomimetic tumor-tissue interfaces. (c) Migration speed before and after crossing the interface. nt – non transmigrated, t – transmigrated. Data are presented as mean ± 95% confidence interval. Statistical significance was tested using a Kruskal-Wallis test. Significance levels were defined as p < 0.05 (*: p < 0.05; **: p < 0.01; ***: p < 0.001). n=17.*

Mean Squared Displacement Analysis of MDA-MB-231 Migration Trajectories

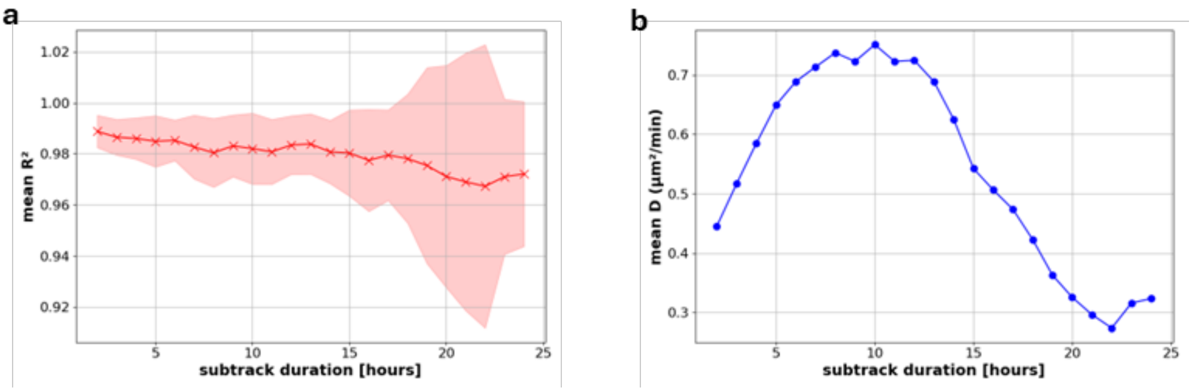

Figure S3: *Determination of maximum Δt for mean squared displacement (MSD) analysis of MDA-MB-231 cell migration in collagen I matrices. (a) Mean R^2 values for linear regression of MSD as a function of different maximum Δt values (including confidence interval of 95%). (b) Mean migration constant D derived from linear regression of MSD using different maximum Δt values ($(\Delta x)^2 = 6D \cdot \Delta t$).*

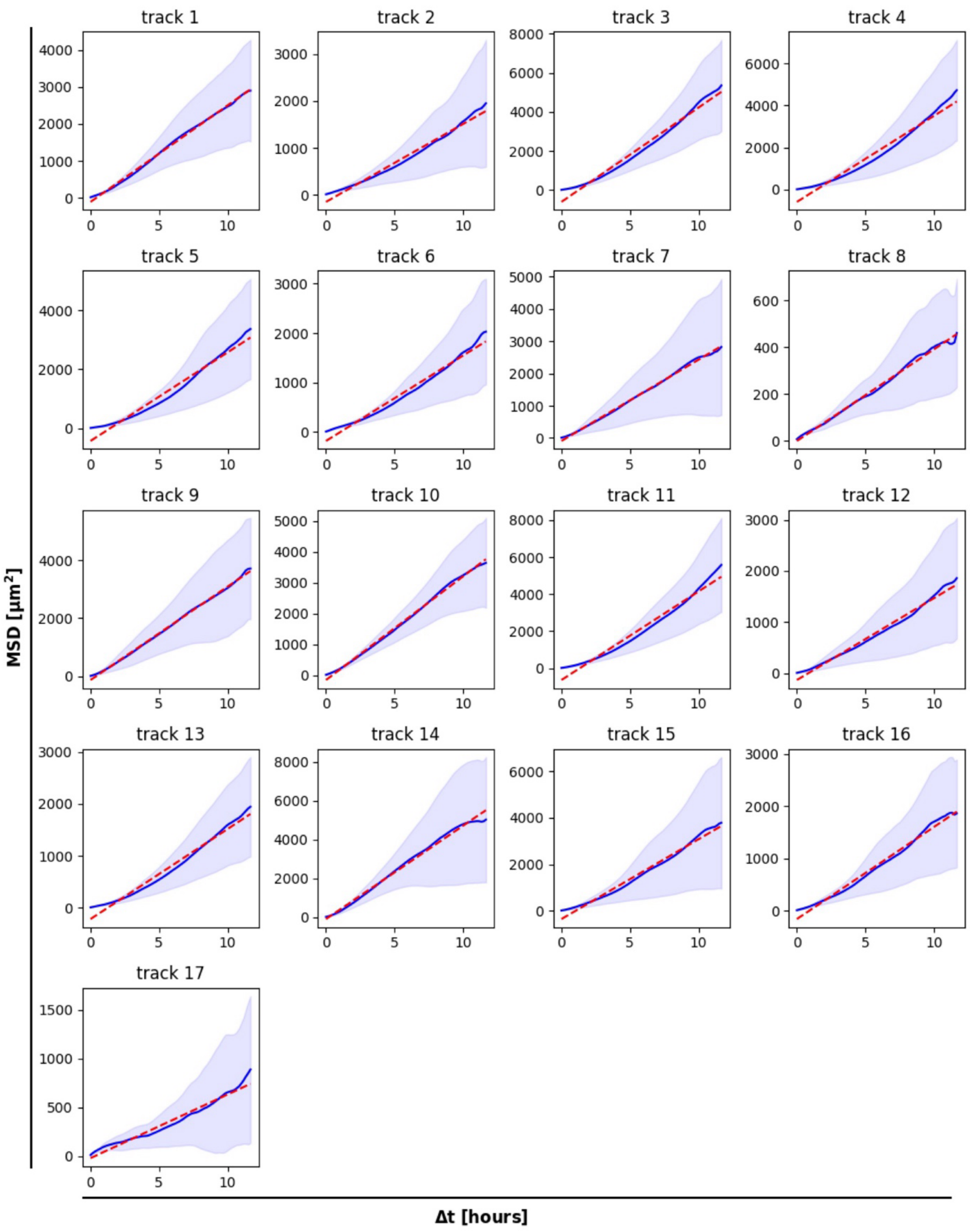

Figure S4: *Mean squared displacement (MSD) analysis of MDA-MB-231 cells in collagen I matrices. MSD analysis was performed for 17 MDA-MB-231 breast cancer cells using a maximum* Δt *of 12 hours. Data are presented as mean ± 95% confidence interval.*

Cell Counting during Transmigration across the Matrix Interface within 24 hours

Section S1: Cell diffusion out of a homogeneously populated half-space with absorbing boundary

Cell transmigration from the dense into the open compartment including a migratory phenotype switch from random to directed migration was described by using an approach of cell diffusion out of a homogeneously populated half-space with an absorbing boundary. The absorbing boundary condition was chosen to describe our observation of a changed migratory phenotype after transmigration with a directed migration away from the interface and no cells migrating back into the dense compartment. Such a situation can be described in one dimension perpendicular to the matrix interface. As a result we reveal the survival function $S(x,t)$ of cells remaining in the dense compartment with:

$$S(x,t) = erf\left(x/\sqrt{4Dt}\right)$$

Integrating over the half-space of the dense compartment for the leaving cells $(1\text{-}S(x,t))$ it results in:

$$N_o = n_d\sqrt{4Dt/\pi}$$

with n_d being the one-dimensional cell density in the dense compartment and N_o the number of expected cells in the open compartment.

With the determined diffusion coefficient of 0.75 µm/s^2 (from MSD analysis) and a volume size in x-direction perpendicular to the matrix interface of 156 µm, we derive for the expected cell number in the open compartment after 24 hours $N_o(24\text{h})$:

$$N_o(24h) = \frac{37\ \mu m}{156\ \mu m} N_d(0h) \ = \ 0.23 \cdot N_d(0h)$$

Hence, 23% of cells found in the dense compartment at 0 hours can be expected to randomly migrate into the dense compartment after 24 hours.

For MDA-MB-231 cells a proliferation constant of 2 within 24 hours is experimentally observed. Hence, a cell doubling has to be roughly assumed for cells leaving the dense compartment or being within the open compartment after transmigration. With this fact we result at the final expectation that 46% of cells in the dense compartment at 0 hours (N_d(0h)) might be found after 24 hours in the open compartment (N_o(24h)).

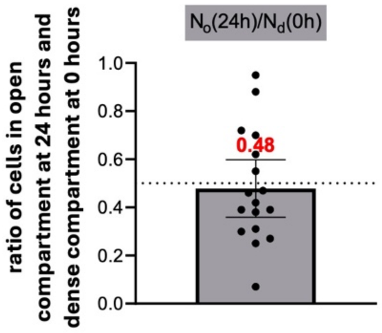

Figure S5: *Cell counting results in dense and open compartments before and after transmigration at time points 0 hours and 24 hours, respectively. Cells were imaged at the d→o matrix interface for 7 days, followed by MSD analysis in 3D to extract the diffusion coefficient D. A volume of 156 µm x 156 µm x 250 µm was set next to the interface in both compartments and cell counts were performed within these volumes at time points 0 hours and 24 hours (in the dense (N_d) and open (N_o) compartment). Ratio of transmigrated cells (N_o(24h) – cells in open compartment after 24 hours) to the cell number at 0 hours in the dense compartment (N_d(0h)). This ratio quantifies the proportion of cells that have migrated from the dense compartment to the open compartment within 24 hours including proliferation within 24 hours. Data are presented as mean ± 95% confidence interval. n=17.*

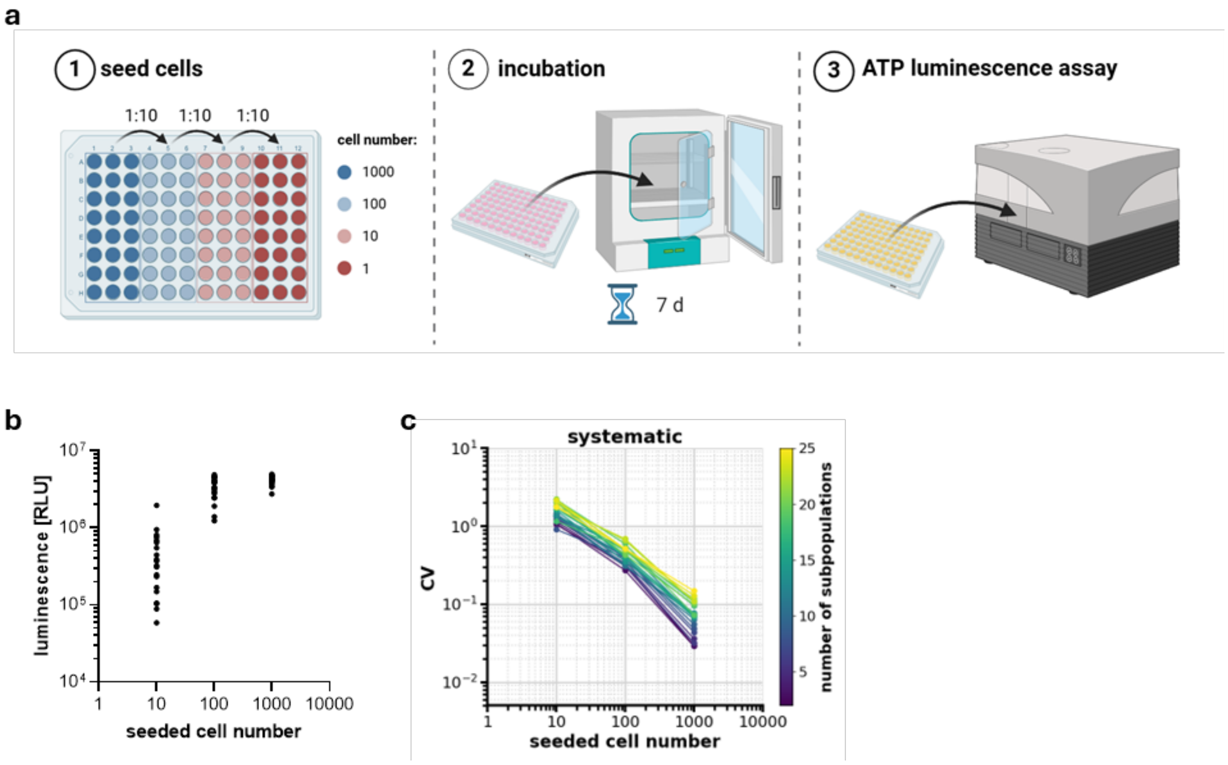

Figure S6: *Assessment of cellular heterogeneity in proliferation using a logarithmic dilution series. (a) Experimental setup for the logarithmic dilution in a 96-well plate. After 7 days of cultivation under standard conditions, the heterogeneity was assessed using an ATP luminescence assay. Created in BioRender. Clemens, C. (2025) https://BioRender.com/2a7ew0y (b) Representative ATP luminescence assay data for two-dimensionally cultivated cells from a standard cell culture flask. (c) Simulation of cellular heterogeneity in proliferation, incorporating a 3% experimental error and testing different numbers of subpopulations.*
